# ALZHEIMER’S DIAGNOSIS AND CARE BY RACIAL AND ETHNIC GROUP IN THE MEDICARE POPULATION

**DOI:** 10.1093/geroni/igae098.3788

**Published:** 2024-12-31

**Authors:** Margaret Hoyt, Melissa Bailey-Taylor, Julie Chandler, Joseph Johnston

**Affiliations:** Eli Lilly and Company, Indianapolis, Indiana, United States; Eli Lilly and Company, Indianapolis, Indiana, United States; Eli Lilly and Company, Indianapolis, Indiana, United States; Eli Lilly and Company, Indianapolis, Indiana, United States

## Abstract

Little is known about differences in healthcare resource utilization (HCRU) among racial/ethnic groups with Alzheimer’s disease (AD). Our study aims to address this gap with an incident AD cohort in Medicare claims data. We identified Medicare patients ≥65 years old with a first (index) AD code (ICD-10 G30*) or medication (galantamine, rivastigmine, donepezil, or memantine) in 2018 or 2019 who were continuously enrolled (gap < 45 days) one year pre- and post-index. Descriptive analyses of HCRU and cost data were stratified by race/ethnicity. Our analysis included 887,984 patients, the majority female (64.2%), with mean (sd) age 80.6 (7.5) years. 72.0% identified as non-Hispanic White, 9.9% non-Hispanic Black, 12.8% Hispanic, and 3.8% Asian. In the 2018-2019 Medicare population, the incidence of Alzheimer’s disease per 100,000 person-years was 1,349 for White, 1,649 Black, 2,003 Hispanic, and 1,500 for Asian patients. Fewer White patients (18.8%) were dual-Medicare/Medicaid eligible than non-White patients (46.0-64.4%), and non-White patients had higher rates of many chronic comorbid illnesses. More White patients had at least one neurologist visit in the year pre-index (39.0%) compared to Black (35.1%), Hispanic (37.5%), and Asian (33.1%) patients. More Black patients had an inpatient admission with AD as the primary diagnosis post-index (11.0%) than White (9.5%), Hispanic (7.6%), and Asian (5.8%) groups, and costs for long-term care and hospice were highest for Black and Hispanic patients, respectively, which could indicate more severe disease. These statistically significant results suggest discrepancies between racial/ethnic groups in the Medicare AD population; further research is needed to understand the differences.

